# *Ulmus parvifolia* Modulates Platelet Functions and Inhibits Thrombus Formation by Regulating Integrin α_IIb_β_3_ and cAMP Signaling

**DOI:** 10.3389/fphar.2020.00698

**Published:** 2020-05-19

**Authors:** Muhammad Irfan, Hyuk-Woo Kwon, Dong-Ha Lee, Jung-Hae Shin, Heung Joo Yuk, Dong-Seon Kim, Seung-Bok Hong, Sung-Dae Kim, Man Hee Rhee

**Affiliations:** ^1^ Laboratory of Physiology and Cell Signaling, College of Veterinary Medicine, Kyungpook National University, Daegu, South Korea; ^2^ Department of Biomedical Laboratory Science, Far East University, Eumseong, South Korea; ^3^ Department of Biomedical Laboratory Science, Molecular Diagnostics Research Institute, Namseoul University, Cheonan, South Korea; ^4^ Herbal Medicine Research Division, Korea Institute of Oriental Medicine, Daejeon, South Korea; ^5^ Department of Clinical Laboratory Science, Chungbuk Health & Science University, Chungbuk, South Korea; ^6^ Research Center, Dongnam Institute of Radiological and Medical Sciences, Busan, South Korea

**Keywords:** *U. parvifolia*, ethnomedicine, platelet, integrin α_IIb_β_3_, cyclic-AMP, VASP^ser157^

## Abstract

**Background:**

The prevalence of cardiovascular diseases (CVDs) is increasing at a high rate, and the available treatment options, sometimes, have complications which necessitates the need to develop safer and efficacious approaches. Ethnomedicinal applications reportedly reduce CVD risk. *Ulmus parvifolia* Jacq. (Ulmaceae) commonly known as Chinese Elm or Lacebark Elm, is native to China, Japan, and Korea. It exhibits anti-inflammatory, antiviral, and anticancer properties, but its anti-platelet properties have not yet been elucidated.

**Purpose:**

To investigate the pharmacological anti-platelet and anti-thrombotic effects of *U. parvifolia* bark extract.

**Study Design and Methods:**

Human and rat washed platelets were prepared; light transmission aggregometry and scanning electron microscopy was performed to assess platelet aggregation and the change in platelet shape, respectively. Intracellular calcium mobilization, ATP release, and thromboxane-B2 production were also measured. Integrin α_IIb_β_3_ activation was analyzed in terms of fibrinogen binding, fibronectin adhesion, and clot retraction. The expression of MAPK, Src, and PI3K/Akt pathway proteins was examined. Cyclic nucleotide signaling pathway was evaluated *via* cAMP elevation and VASP phosphorylation. Anti-thrombotic activity of the extract was evaluated *in vivo* using an arteriovenous shunt rat model, whereas its effect on hemostasis in mice was assessed *via* bleeding time assay.

**Results:**

*U. parvifolia* extract significantly inhibited human and rat platelet aggregation in a dose-dependent manner along with inhibition of calcium mobilization, dense granule secretion, and TxB2 production. Integrin α_IIb_β_3_ mediated inside-out and outside-in signaling events, as evidenced by the inhibition of fibrinogen binding, fibronectin adhesion, and clot retraction. The extract significantly reduced phosphorylation of Src, MAPK (ERK, JNK, and p38^MAPK^), and PI3K/Akt pathway proteins. Cyclic-AMP levels were elevated in *U. parvifolia-*treated platelets, while PKAαβγ and VASP^ser157^ phosphorylation was enhanced. *U. parvifolia* reduced thrombus weight in rats and moderately increased bleeding time in mice.

**Conclusion:**

*U. parvifolia* modulates platelet responses and inhibit thrombus formation by regulating integrin α_IIb_β_3_ mediated inside-out and outside-in signaling events and cAMP signaling pathway.

## Introduction

Cardiovascular diseases (CVDs) are considered a leading cause of death worldwide. In the United States, one in seven and one in nine deaths occurred owing to coronary heart disease and heart failure, respectively, in 2013 (Mozaffarian et al., 2016). World Health Organization stated that CVD accounted for 30% of all deaths in 2005, and in Europe, it remains the primary cause of 42% mortalities in men and 52% in women (Nichols et al., 2013). Following vascular injury, platelets play a crucial role in maintaining hemostasis and preventing blood loss *via* thrombus formation; however, pathophysiological hyper-activation of platelets is the major cause underlying thrombotic complications, which contribute toward the development of cardiovascular ailments including atherosclerosis, coronary heart disease, stroke, and heart attack (Andrews and Berndt, 2004).

Pharmacological platelet suppression effectively reduces thrombotic events, and many clinical drugs are available for treating and preventing CVD. However, side effects and complications (*e.g.*, gastric bleeding in case of aspirin and sometimes, thrombocytopenia or aplastic anemia in case of clopidogrel) caused by those drugs may outweigh their benefits (Barrett et al., 2008), while a significant part of population is resistant to most commonly used anti-platelet agents *i.e.*, aspirin and clopidogrel (Wang et al., 2006; Ferguson et al., 2008) which necessitate the development of alternative preventive and therapeutic approaches with no or minimal drug-associated complications. Besides anti-platelet drug-based treatment options for thrombotic disorders and CVD-related complications, there is increasing focus on the use of natural products and their bioactive natural compounds including ethnomedicinal applications for CVD treatment and prevention (Badimon et al., 2010; Kim, 2018); similarly, innumerable natural products, including traditional Mediterranean diet and medicinal plants, have also been proven effective with regard to their cardioprotective and anti-platelet effects in the primary and secondary prevention of CVD (Estruch et al., 2013; Rastogi et al., 2016; Irfan et al., 2018c; Shin et al., 2019).

The genus *Ulmus* includes several species which produce fine wood, medicinal products, and edible fruit. Several *Ulmus* species reportedly possess anti-oxidative (Jung et al., 2010) and anti-platelet (Yang et al., 2013) properties. *Ulmus parvifolia* Jacq., commonly known as the Chinese Elm or Lacebark Elm, is native to Korea, Japan, and China, reportedly possesses anti-inflammatory (Mina et al., 2016), anti-allergic (Kim et al., 2016), anti-viral, and anti-cancer (Hamed et al., 2015) properties. While investigating newer and safer efficacious ethnomedicinal products, we recognized the medicinal properties of *U. parvifolia* bark. Here, we aimed to evaluate anti-platelet and anti-thrombotic effects of *U. parvifolia* bark ethanol extract on human and rat platelets and explored its pharmacological composition.

## Materials and Methods

### Extraction and Identification of Active Compounds

Bark of *U. parvifolia* was obtained from National Institute of Horticultural and Herbal Science (Jeollabuk-do, Republic of Korea). *U. parvifolia* bark was grounded and extracted with 70% ethanol at 80°C for 3 h, filtered with Whatman™ filter paper (GE Healthcare, PA, USA), condensed in a rotary evaporator (Rotavapor^®^ R-100; B.U.CHI Labortechnik, Switzerland), and lyophilized to obtain powdered extract. The powder was stored at −30°C for further use in experiments.

#### Identification of the Chemical Constituents

Ultra-performance lipid chromatography (UPLC) system (Waters Corp., Milford, MA, USA), equipped with a binary solvent delivery system, an auto-sampler, and a UV detector, was used for performing chromatographic separation to identify the chemical constituents of *U. parvifolia* extract as previously described (Jung et al., 2010). Briefly, aliquots (2.0 μl) of each sample were injected into a BEH C_18_ column (2.1 × 100 mm, 1.7 μm) at a flow rate of 0.4 ml/min and eluted using a chromatographic gradient of two mobile phases (A: water containing 0.1% formic acid; B: acetonitrile containing 0.1% formic acid). A linear gradient was optimized as follows: 0 min, 5% B; 0–8 min, 5–15% B; 8–11 min, 15–80% B; 11–12 min, 80–100% B; 12–13.3 min, 100% B; 13.4–15 min, back to 5% B.

#### Quantitative Analysis of Identified Compounds

Two main peaks (1 and 2) for catechins were quantified using UV detector at 280 nm wavelength and were calculated using a standard curve obtained from an authentic catechin standards. Standard calibration curves were plotted over a concentration range of 0.0012.5–0.2 mg/ml for ﬁve different concentrations of catechin and catechin-7-*O*-β-_D_-apiofuranoside (*r* ^2^ > 0.998). The amount of compound was ﬁnally expressed as mg/g of ethanol extract.

### Animals

Sprague–Dawley rats weighing 220–240 g and 7-week-old C57BL/6J male mice weighing 20–22 g were purchased from Orient Co. (Seoul, Republic of Korea); prior to the experiment, they were acclimatized for 1 week in a special animal room maintained at 23 ± 2°C and 50 ± 10% humidity under 12-h light/dark cycles. Experiments were conducted according to IACUC guidelines, and experimental protocols were approved by the Ethics Committee of College of Veterinary Medicine, Kyungpook National University, Daegu, Korea (Permit number: 2017-0014).

### Preparation of Washed Human and Rat Platelets

Human platelet-rich plasma (PRP) collected from healthy volunteers who provided informed consent was obtained from the Korean Red Cross Blood Center (KRBC, Changwon, Korea), and its experimental use was approved by KRBC and the Korea National Institute for Bioethics Policy Public Institutional Review Board (PIRB17-1019-03). Washed human and rat platelets were prepared as previously described (Irfan et al., 2018a).

### Platelet Aggregation Assay and Scanning Electron Microscope (SEM) Analysis

To assess platelet aggregation, the standard procedure of light-transmission aggregometry was performed using a Chrono-log aggregometer (Havertown, PA, USA), as previously described (Irfan et al., 2018b). Briefly, washed platelets were pre-incubated with various concentrations of either *U. parvifolia* extract or vehicle (DMSO) for 1 min in the presence of 1 mM calcium chloride (CaCl_2_), followed by stimulation with various agonists (Collagen, ADP, or thrombin) for 5 min with continuous stirring at 37°C. DMSO concentration was maintained at <0.1%.

A field emission SEM was used to assess platelet shape change and aggregation by obtaining ultrastructure images as previously described (Irfan et al., 2018a).

### Immunoblotting

Washed platelets were pre-incubated with various concentrations of *U. parvifolia* extract along with 1 mM CaCl_2_ for 1 min at 37°C and then stimulated with collagen for 5 min under continuous stirring. Platelet aggregation was terminated by adding lysis buffer (PRO-PREP; iNtRON Biotechnology, Seoul, Korea), and protein concentration was estimated using BCS assay (PRO-MEASURE; iNtRON Biotechnology). Total platelet proteins were separated in a 10% SDS-PAGE and transferred to PVDF membranes. Membranes were blocked with 5% skim milk, probed with respective antibodies (*i.e.*, Src, phospho-Src, ERK, phospho-ERK, JNK, phospho/JNK, p38^MAPK^, phospho-p38^MAPK^, phospho-PI3K, PI3K, Akt, phospho-Akt, VASP, phospho-VASP^ser157^, PKAαβγ, and β-actin) and visualized using enhanced chemiluminescence.

### Arteriovenous Shunt Model

The anti-thrombotic activity of *U. parvifolia* extract was assessed in a rat extracorporeal shunt model as previously described (Irfan et al., 2018c). Rats were orally administered with the vehicle, *U. parvifolia* extract, or ASA once daily for 3 days. Two hours after the last administration, rats were anesthetized and shunt was placed for 15 min after initiating extracorporeal circulation. Subsequently, blood flow was stopped and the thrombus formed was weighed.

### *In Vivo* Bleeding Assay

Male mice were divided into three treatment groups (n = 5, each). They were intraperitoneally administered with saline, ASA, or *U. parvifolia* extract once daily for 3 days. One hour after the last administration, mice were anaesthetized, and tail bleeding assay was performed as previously described (Irfan et al., 2018b).

### Statistical Analysis

Data were analyzed by one-way analysis of variance (ANOVA), followed by measurement of statistically significant differences using Dunnett's *post-hoc* test (SAS Institute Inc., Cary, NC, USA). All data are presented as the mean ± standard deviation (SD). A *p* value of ≤0.05 was considered statistically significant.

Detailed description on; chemicals and reagent sources, preparation of washed human and rat platelets, scanning electron microscope (SEM) analysis, ATP release assay, measurement of [Ca^2+^]*_i_* mobilization, flow cytometry, fibronectin adhesion assay, clot retraction, measurement of cyclic-AMP, AV-shunt model, and *in vivo* bleeding assay has been included in **Supplementary Material**.

## Results

### *U. parvifolia* Inhibits Agonist-Induced Human and Rat Platelet Aggregation

**Figure 1:**
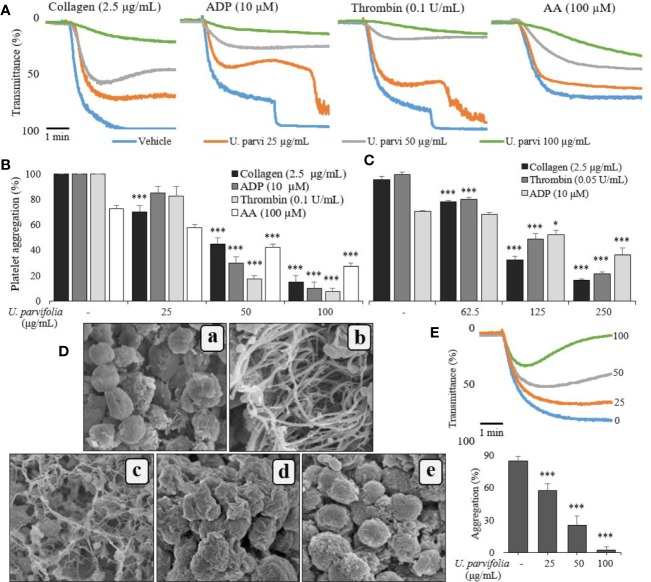
*U. parvifolia* inhibits agonist-induced human and rat platelet aggregation. Collagen-, ADP-, arachidonic acid (AA), or thrombin-stimulated washed platelets **(A, B, D, E**, from rats; **C**, from humans) pre-treated with vehicle or various *U. parvifolia* extract concentrations in the presence of 1-mM CaCl_2_. **(D)** Representative SEM images (5,000×) of collagen (2.5 µg/ml)-stimulated platelets pre-treated with vehicle or various *U. parvifolia* extract concentrations [(a) Resting, (b) Vehicle, (c) 25 µg/ml, (d) 50 µg/ml, (e) 100 µg/ml]. **(E)** Rat PRP was incubated with vehicle or various concentrations of extract in the presence of 10-mM CaCl_2_ for 1 min and then stimulated with ADP (25 µM) for 5 min. The graphs show mean ± SD values from at least four independent experiments. *p < 0.05 and ***p < 0.001 *versus* control.

Initial screening confirmed that *U. parvifolia* was effective against human and rat platelet aggregation induced by several agonists, including collagen, ADP, AA, and thrombin. **Figures 1A, B** show that *U. parvifolia* significantly inhibited agonist-induced aggregation in a dose-dependent manner in rat platelets; similar inhibitory results were obtained in agonist-induced aggregation in human platelets (**Figure 1C**). The extract also potently inhibited ADP-induced aggregation measured in rat PRP, in a dose-dependent manner (**Figure 1E**). We examined the effect of *U. parvifolia* extract on platelet shape change and aggregation using SEM. We found a dose-dependent inhibition of collagen-induced platelet shape change and aggregation in *U. parvifolia*-treated rat platelets compared with that in vehicle-treated platelets (**Figure 1D**).

### *U. parvifolia* Reduces [Ca^2+^]_i_ Mobilization, Dense Granule Secretion, and Thromboxane-B2 Production

**Figure 2:**
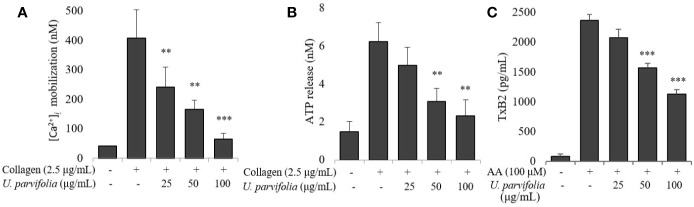
*U. parvifolia* inhibits [Ca^2+^]*_i_* mobilization and reduces ATP release and TxB2 production. **(A)** Fura 2/AM-loaded rat platelets pre-treated with vehicle or various *U. parvifolia* extract concentrations and stimulated with collagen (2.5 µg/ml) for 3 min. Assessment of ATP concentration **(B)**, and thromboxane-B2 production **(C)** was done in supernatant of stimulated washed platelets suspension pre-treated with vehicle or various *U. parvifolia* extract concentrations and stimulated with collagen for 5 min on a luminometer or TxB2 ELISA kit, respectively. Results are represented as mean ± SD values from at least four independent experiments. **p < 0.01 and ***p < 0.001 *versus* control.

We assessed the effect of *U. parvifolia* extract on collagen-stimulated platelet intracellular calcium ion ([Ca^2+^]*_i_*) mobilization, ATP release, and thromboxane production; and found its significant and dose-dependent inhibitory activity against [Ca^2+^]*_i_* mobilization while it also markedly reduced ATP and thromboxane-B2 release (**Figure 2**).

### *U. parvifolia* Attenuates Integrin α_IIb_β_3_-Mediated Inside-Out and Outside-In Signaling

**Figure 3:**
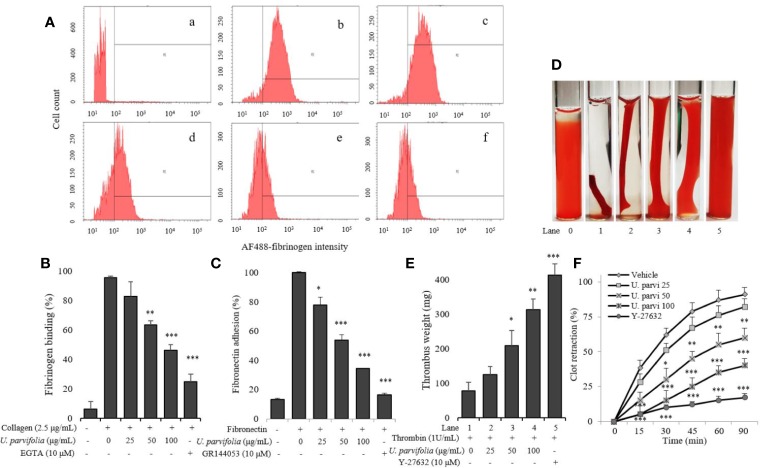
*U. parvifolia* inhibits integrin α_IIb_β_3_-mediated inside-out and outside-in signaling. **(A, B)** Flow cytometric measurements of fibrinogen binding in platelets treated with vehicle, various *U. parvifolia* extract concentrations or EGTA [(a) Resting, (b) Vehicle, (c) 25 µg/ml, (d) 50 µg/ml, and (e) 100 µg/ml, (f) 10 µM EGTA)] and stimulated with collagen (b-f). **(C)** Results of fibronectin adhesion assay, which was performed using an assay kit according to the manufacturer's protocol and by following the procedure described in the methods section. **(D)** *In vitro* effect of *U. parvifolia* extract on clot retraction for 2 h at room temperature after thrombin addition and photographed with 15 min intervals. Representative images of clot retraction at 90 min after thrombin addition in the presence and absence of *U. parvifolia* extract. Y-27632 (ROCK inhibitor) was used as a control. **(F)** Kinetics of clot retraction were measured by Image-J software and clot surface areas were plotted as a percentage of retraction. Bar graphs summarizing the inhibitory effect of *U. parvifolia* extract on fibrinogen binding to integrin α_IIb_β_3_ **(B)**, fibronectin adhesion **(C)**, clot retraction **(E)**, and kinetics of clot retraction **(F)**. Results are shown as mean ± SD values from at least four independent experiments. *p < 0.05, **p < 0.01, and ***p < 0.001 *versus* control.

We examined whether various concentrations of *U. parvifolia* extract modulate collagen-stimulated platelet integrin signaling, and found that they significantly attenuated fibrinogen binding to integrin α_IIb_β_3_ in a dose-dependent manner (**Figures 3A, B**). We also examined inhibitory activity of *U. parvifolia* extract on fibronectin adhesion and found that it significantly and dose-dependently reduced platelet adhesion on fibronectin-coated surface compared with vehicle control (**Figure 3C**). We also found that *U. parvifolia* extract significantly reduced clot retraction in a dose-dependent manner (**Figures 3D–F**).

### *U. parvifolia* Attenuates MAPK, Src, and PI3K/Akt Phosphorylation

**Figure 4:**
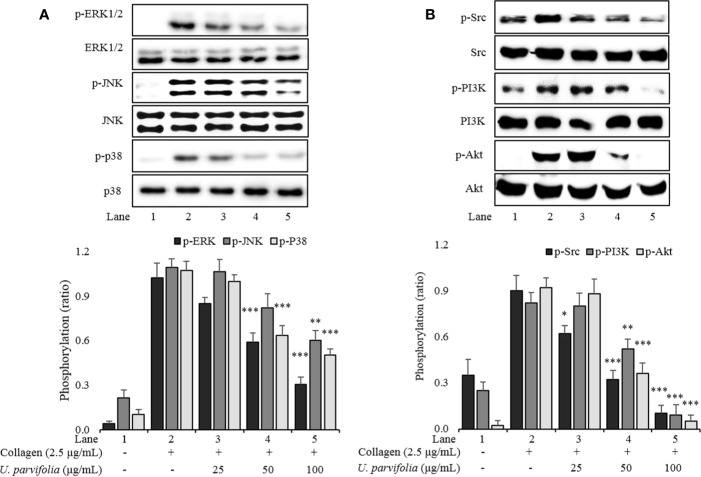
*U. parvifolia* attenuates phosphorylation levels of MAPKs **(A)**, Src, and PI3K/Akt **(B)**. Immunoblotting was conducted to analyze the phosphorylation of signaling molecules extracted from the lysates of collagen-stimulated washed platelets that were pre-treated with either *U. parvifolia* extract or vehicle. Representative immunoblot images and data (mean ± SD) from at least four independent experiments are shown. *p < 0.05, **p < 0.01, and ***p < 0.001 *versus* agonist-treated group.

We explored the underlying inhibitory mechanism of the extract, and our results revealed that *U. parvifolia* extract significantly inhibited collagen-mediated GPVI downstream signaling by attenuating the phosphorylation of ERK, JNK, p38^MAPK^, Src, and PI3K/Akt pathway molecules (**Figure 4**).

### *U. parvifolia* Elevates cAMP Levels and Enhances VASP and PKAαβγ Phosphorylation

**Figure 5:**
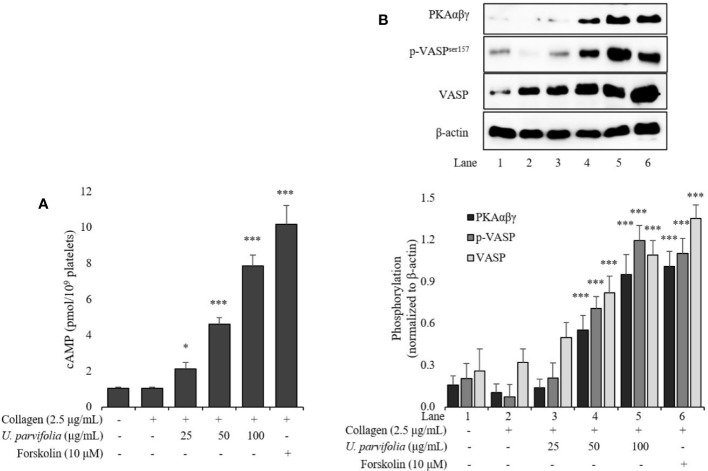
*U. parvifolia* elevates cAMP production and enhances VASP and PKAαβγ phosphorylation. **(A)** cAMP immunoassay performed for collagen-stimulated washed platelets pre-treated with various *U. parvifolia* extract concentrations, vehicle, or forskolin. **(B)** Immunoblotting analysis conducted for the phosphorylation of total VASP, p-VASP^ser157^, and PKAαβγ extracted from platelets. Representative immunoblot images and graphs plotted for the data (mean ± SD values) obtained from at least four independent experiments are shown. *p < 0.05 and ***p < 0.001 *versus* agonist-treated group.

We assessed intracellular cAMP levels in collagen-stimulated platelets which were markedly amplified owing to treatment with ascending concentrations of *U. parvifolia* extract (**Figure 5A**). Similarly, phosphorylation of not only VASP but also phospho-VASP^ser157^ (a preferred site by PKA) was significantly enhanced in a dose-dependent manner in *U. parvifolia*-treated platelets. Furthermore, to evaluate whether the observed effect is mediated *via* cAMP–PKA–VASP^ser157^-dependent pathway, we assessed PKAαβγ phosphorylation; it was also found to be significantly increased in a dose-dependent manner (**Figure 5B**).

### *U. parvifolia* Prevents Thrombosis and Regulates Hemostasis

**Figure 6:**
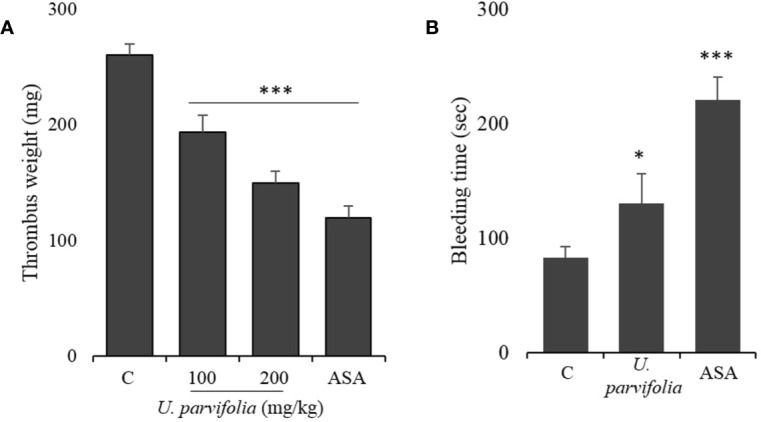
*U. parvifolia* inhibits thrombus formation and modulates hemostasis. **(A)** Evaluation of *in vivo* anti-thrombotic activity and determination of thrombus weight in AV shunt model of rats that were orally administered with saline, *U. parvifolia* extract (100–200 mg/kg), or ASA (50 mg/kg). **(B)** Results of tail bleeding assay for homeostasis measurement in mice administered with *U. parvifolia* extract (200 mg/kg), ASA (50 mg/kg), or saline (n = 5 in each group). Graph shows mean ± SD values from at least five independent experiments performed. *p < 0.05; ***p < 0.001 *versus* control.

**Figure 6A** shows that *U. parvifolia* extract significantly reduced thrombus weight in a dose-dependent manner compared with vehicle control. Similarly, our results of tail bleeding assay in mice also revealed that *U. parvifolia* extract moderately increased bleeding time compared to vehicle control while time to cessation of bleeding was greatly increased in ASA group (**Figure 6B**).

### Chemical Constituents of *U. parvifolia* Extract

**Figure 7:**
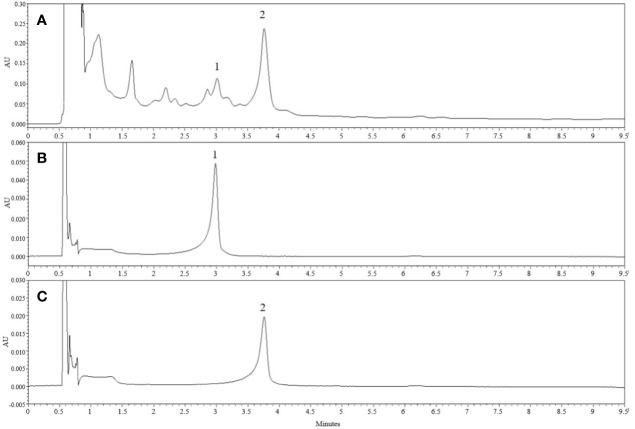
Chemical constituents of *U. parvifolia* extract. **(A)** The UPLC chromatogram of EtOH extract of *U. parvifolia* was detected at 280 nm UV. **(B, C)** Chromatograms of a standard solution. Peaks 1 ((+)-catechin) and 2 (catechin-7-O-β-D-apiofuranoside).

UPLC results revealed marker compounds in the extract *i.e.*, catechin (tR = 2.98 min) and catechin-7-*O*-β-_D_-apiofuranoside (tR = 3.651 min) (**Figure 7**). Quantitative analysis of the given extract revealed that catechin and catechin-7-*O*-β-_D_-apiofuranoside were present at concentrations of 6.14 and 156.3 mg/g, respectively.

## Discussion

Here, we evaluated anti-platelet and anti-thrombotic effects of *U. parvifolia* bark ethanol extract and determined its potential pharmacological properties involved in the modulation of platelet functions which may be attributed to its phenolic constituents, especially catechins.

**Figure 8:**
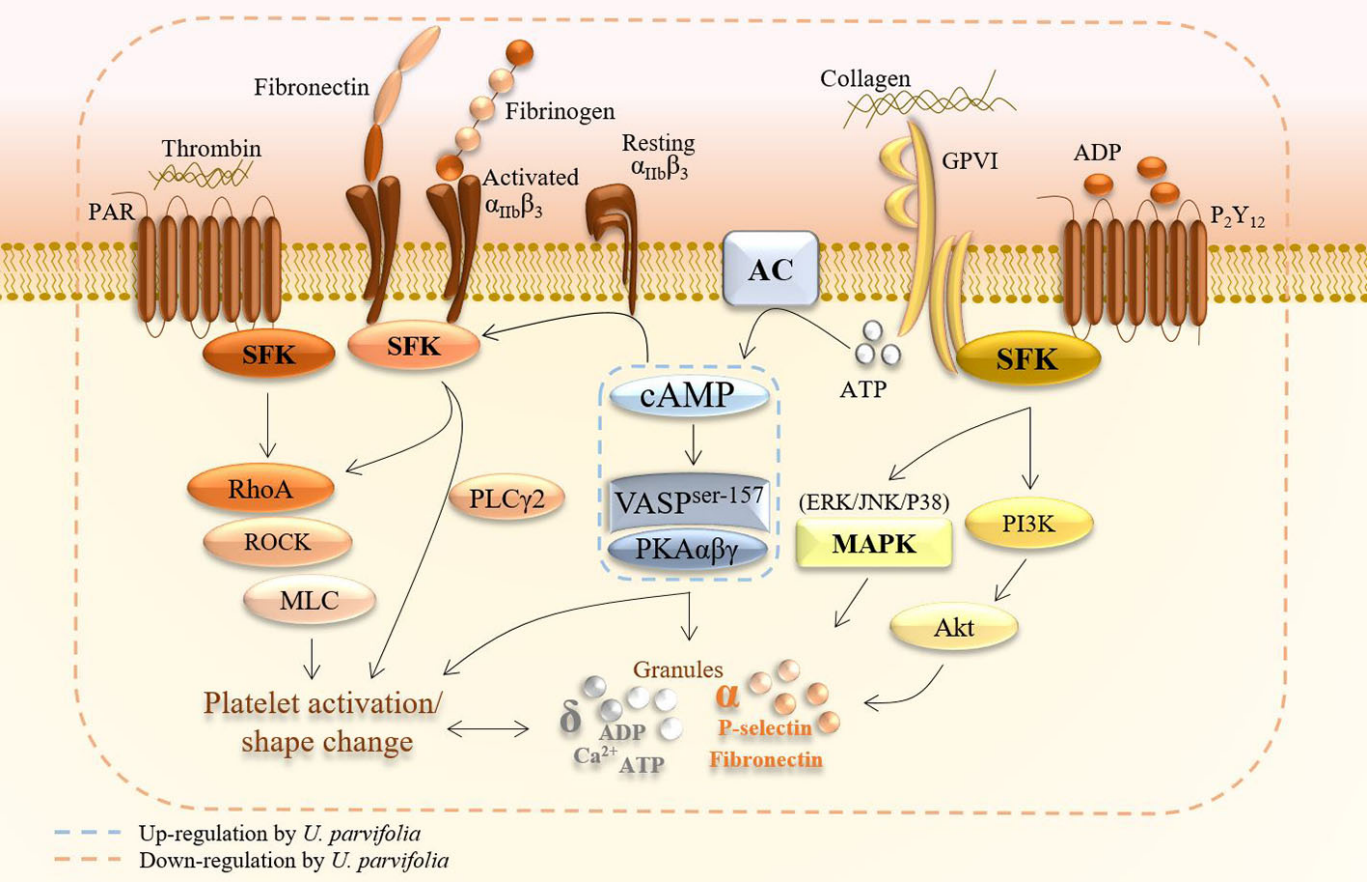
A schematic summary of inhibitory effects of *U. parvifolia* extract on platelet intracellular signaling pathway.

Collagen, thrombin, and ADP are agonists that induce strong platelet aggregation by triggering downstream signaling events, including granule secretion, *via* activation of GPVI, proteinase-activated receptor (PAR), and P2Y12 receptor signaling pathways, respectively. These platelet-signaling events are subdivided into (i) early receptor signaling triggered by agonists or adhesive stimulants; (ii) merging of common pathways and amplification of signaling; (iii) inside-out signaling which causes conformational change of integrin α_IIb_β_3_ structure to allow fibrinogen binding and early phase of platelet adhesion; and (iv) outside-in signaling which augments late phases of adhesion and clot retraction. Moreover, platelets contain dense (δ) granules, including Ca^2+^, ATP, and serotonin, as well as alpha (α) granules packed with adhesive ligands including fibrinogen, fibronectin, and P-selectin. Secretion of these granules upon platelet activation further enhances platelet adhesion, shape change, and aggregation (**Figure 8**) (Estevez and Du, 2017; Irfan et al., 2020). The unceasing change in shape, which results in full activation of platelets, can be best observed under SEM. Our initial screening results revealed that *U. parvifolia* extract had strong inhibitory effects against the above mentioned agonists, and it significantly inhibited platelet aggregation and shape change in a dose-dependent manner; these findings indicated that the extract exerts strong anti-platelet activity *via* inhibition of several platelet-signaling pathways, as detailed in **Figure 8**. To examine the effects on physiological condition of platelets, we used rat PRP and found that the extract potently inhibited ADP-induced platelet aggregation; indicating that constituents of extract have strong ability to modulate platelet responses and aggregation. COX-1 inhibitor like aspirin and antagonists of the P2Y12 for ADP like clopidogrel and ticagrelor are currently used in coronary interventions and prevention of cardiovascular thrombotic events which are sometimes, impeded by side effects or resistant to some patients (Cattaneo, 2011). Our results show that *U. parvifolia* extract with several pharmacological constituents could be a potent natural candidate to deal with platelet-related disorders.

To investigate the underlying mechanism, we further examined intracellular [Ca^2+^]*_i_* mobilization, dense granule secretion, and thromboxane production. We found that our extract markedly inhibited [Ca^2+^]*_i_* mobilization, ATP secretion, and thromboxane-B2 production. Similarly, significant inhibition of fibrinogen binding to integrin α_IIb_β_3_ and fibronectin adhesion was observed, indicating that *U. parvifolia* extract modulates integrin α_IIb_β_3_-mediated inside-out signaling.

When fibrinogen binds to α_IIb_β_3_, it transduces signal into the cell (termed as outside-in signaling), which further enhances platelet adhesion and spreading, thrombus formation, and clot retraction (Irfan et al., 2018a). Similarly, fibronectin is another adhesive ligand that stabilizes thrombus formation in the vasculature. It binds to integrin α_IIb_β_3_ and augments platelet aggregation by developing cohesive aggregates (Pankov and Yamada, 2002). GTPases regulate platelet adhesion, shape change, and clot retraction through actin cytoskeletal changes. Rho kinases (ROCKs) are downstream regulators which mediate RhoA-stimulated actin cytoskeletal changes *via* myosin light chain phosphorylation. The role of Rho kinase in facilitating clot retraction has been previously described using Rho kinase or ROCK inhibitor (Y-27632) (Liao et al., 2007). Among SFKs, Src kinase is predominantly expressed in platelets and plays a vital role in integrin α_IIb_β_3_-mediated signaling and is reportedly also involved in clot retraction (Senis et al., 2014). *U. parvifolia* extract significantly inhibited clot retraction and reduced Src phosphorylation. This result suggested that *U. parvifolia* extract modulated integrin α_IIb_β_3_-mediated outside-in signaling by inhibiting Rho kinase and Src kinase and consequently inhibiting platelet activation. Antagonists of glycoprotein IIb/IIIa (α_IIb_β_3_) such as abciximab and eptifibatide are used to reduce occlusive arterial events in patients with atherosclerosis (Cattaneo, 2011). Our results show that the extract and its constituents could be a great natural alternate to be considered in clinical interventions.

MAPKs (ERK, JNK, and p38^MAPK^) are highly expressed in platelets and become activated when platelets encounter any potential agonists; phosphorylation of these proteins triggers granule secretion which further enhances platelet aggregation (Adam et al., 2008). Src family kinases (SFKs) participate in early signaling of platelet activation and play a central role in mediating platelet functions. Moreover, MAPK and PI3K/Akt are downstream effectors of SFK and play critical roles in platelet activation by influencing calcium mobilization, granule secretion, and platelet aggregation (Senis et al., 2014). Here, our *U. parvifolia* extract impeded these molecules and significantly reduced the phosphorylation of MAPK and PI3K/Akt, indicating its possible inhibitory mechanism against platelet functions.

Cyclic-AMP and cyclic nucleotide-dependent protein kinase are suppressed in activated platelets, whereas elevated intracellular cAMP levels inhibit platelet activation and aggregation (Li et al., 2003). Actin dynamics are relegated by vasodilator-stimulated phosphoprotein (VASP) which is a substrate of cyclic nucleotide (cAMP/cGMP)-dependent protein kinases (i.e., PKA/PKG); stimulation of these kinases augment VASP phosphorylation and inhibit platelet activation and aggregation. Elevation of cAMP levels reportedly inhibits platelet activation by activation of VASP *via* specifically increased phosphorylation of VASP^ser157^ (Ok et al., 2012). VASP ^ser157^ acts as a substrate for cAMP-dependent PKA; its stimulation inhibits platelet activation *via* modulation of platelet secretion and adhesion, and its phosphorylation also hinders integrin α_IIb_β_3_-mediated signaling, thereby inhibiting platelet aggregation (Wentworth et al., 2006). Here, *U. parvifolia* extract inhibited platelet activation by upregulating cAMP-PKA-VASP^ser157^ pathway. **Figure 8** summarizes the effects of *U. parvifolia* on platelet intracellular signaling.

AV-shunt model is widely used for assessing anti-thrombotic activity (Endale et al., 2012), and well established to assess *in vivo* anti-thrombotic effects. Umetsu and Sanai (Umetsu and Sanai, 1978) have stated that the shunt comprises activated platelets, fibrin, and trapped erythrocytes and that its formation can be attenuated by anti-platelet agents. A similar thrombus formation occurs in coronary arteries following heart attack or myocardial infarction (Silvain et al., 2011). Our results revealed that as opposed to treatment with vehicle, treatment with *U. parvifolia* moderately increased bleeding time in mice and a substantially reduced thrombus formation in rats *via* inhibition of platelet activation.

UPLC is a powerful tool to identify and characterize the chemical profiles of natural products (Liu et al., 2013). Catechins are polyphenolic phytochemicals exhibiting several biological activities in the human body, potentially in the treatment and prevention of cardiovascular ailments through modulation of blood lipid metabolism, cardioprotective effects including vascular endothelium protection and stabilizing blood pressure (Shaterzadeh-Yazdi et al., 2017), while another study has also summarized the beneficial effects of catechins on cardiovascular system (Braicu et al., 2013). Most of the *Ulmus* species are reported to possess pharmacological properties including antiplatelet effects; due to their polyphenolic contents such as catechin, epicatechin, catechin-7-*O*-β-_D_-apiofuranoside, and catechin-7-*O*-β-_D_-xylopyranoside (Jung et al., 2010; Yang et al., 2013; Park et al., 2020). Among these catechins, catechin-7-*O*-β-_D_-apiofuranoside has been also known for its anti-oxidant, anti-inflammatory and anti-fibrotic properties (MinHee et al., 2003; Jung et al., 2010; Kwon et al., 2011; Park et al., 2020). Previous studies have shown that catechins inhibit platelet activity by regulating calcium mobilization (Kang et al., 2001), impeding several signaling kinases including ERK and p38^MAPK^ (Lill et al., 2003), increasing cAMP levels, and enhancing VASP^ser157^ phosphorylation in platelets (Ok et al., 2012); they also inhibit ERK, JNK, p38^MAPK^, and Akt activation in vascular smooth muscles and endothelial cells. Our UPLC results suggest that the extract predominantly contains catechins, which are most probably responsible for the inhibition of platelet activation and thrombus formation *via* regulation of MAPK pathway and cAMP signaling.

## Conclusion

*U. parvifolia* substantially inhibits agonist-stimulated platelet aggregation, [Ca^2+^]*_i_* mobilization, dense granule secretion, fibrinogen binding, and fibronectin adhesion as well as reduces clot retraction, which in turn inhibit integrin α_IIb_β_3_-mediated inside-out and outside-in signaling. It also attenuates the phosphorylation of Src, MAPK's, and PI3K/Akt-signaling molecules; elevates cyclic-AMP levels; enhances phosphorylation of VASP^ser157^ and PKAαβγ; and inhibits *in vivo* thrombus formation. Taken together, we conclude that *U. parvifolia* modulates platelet functions and inhibits thrombus formation by regulating integrin α_IIb_β_3_ and cyclic nucleotide signaling; thus, it is a potential candidate for preventing and treating platelet-related CVDs.

## Data Availability Statement

All datasets generated for this study are included in the article/**Supplementary Material**.

## Ethics Statement

The studies involving human participants were reviewed and approved by Korean Red Cross Blood Center and the Korea National Institute for Bioethics Policy Public Institutional Review Board (PIRB17-1019-03). Written informed consent for participation was not required for this study in accordance with the national legislation and the institutional requirements. The animal study was reviewed and approved by Ethics Committee of College of Veterinary Medicine, Kyungpook National University, Daegu, Korea (Permit number: 2017-0014).

## Author Contributions

MI and MR designed and conceptualized the study. MI performed experiments, analyzed data, and wrote manuscript. H-WK, D-HL, J-HS, HY, D-SK, S-BH, and S-DK performed partial experiments and analyzed data. MI critically revised the manuscript. MR and S-DK supervised the research work. All authors read and approved the final manuscript.

## Funding

This research was supported by the National Research Foundation of Korea (2018R1D1A1A09083797).

## Conflict of Interest

The authors declare that the research was conducted in the absence of any commercial or financial relationships that could be construed as a potential conflict of interest.

## Abbreviations

AA, Arachidonic acid; ACD, Acid-citrate-dextrose; ADP, Adenosine diphosphate ; AV-shunt, Arteriovenous shunt; Akt, Protein kinase B; DMSO, Dimethyl sulfoxide ; ERK, Extracellular signal-regulated kinase; GPVI, Glycoprotein VI; JNK, c-Jun N-terminal kinase; MAPK, Mitogen-activated protein kinase; PAR, Proteinase-activated receptor; PI3K, Phosphatidylinositol 3-kinases; PKA, Protein kinase A; PRP, Platelet-rich plasma ; Rho-A, Rho kinase A; SEM, Scanning electron microscope ; SFK, Src family kinase; TxB2, Thromboxane-B2; VASP, Vasodilator-stimulated phosphoprotein.
