# Supplementary material for: Ulmus parvifolia Modulates Platelet Functions and Inhibits Thrombus Formation by Regulating Integrin αIIbβ3 and cAMP Signaling
Source: Front Pharmacol. 2020 May 19;11:698. doi: 10.3389/fphar.2020.00698 (PMC7248206; doi:10.3389/fphar.2020.00698)
Supplement: Supplementary file 1 [file DataSheet_1.docx]

**Supplementary Material**

***Ulmus parvifolia* modulates platelet functions and inhibits thrombus formation by regulating integrin α_IIb_β_3_ and cAMP signaling**

**Muhammad Irfan^1^, Hyuk-Woo Kwon^2^, Dong-Ha Lee^3^, Jung-Hae Shin^2^, Heung Joo Yuk^4^, Dong-Seon Kim^4^, Seung-Bok Hong^5^, Sung-Dae Kim^6^, and Man Hee Rhee^1,*^**

^1^ Laboratory of Physiology and Cell Signaling, College of Veterinary Medicine, Kyungpook National University, Daegu 41566, Republic of Korea

^2^ Department of Biomedical Laboratory Science, Far East University, Eumseong 27601, Republic of Korea

^3^ Department of Biomedical Laboratory Science; and Molecular Diagnostics Research Institute, Namseoul University, Cheonan 31020, Republic of Korea

^4^ Herbal Medicine Research Division, Korea Institute of Oriental Medicine, Daejeon 34054, Republic of Korea

^5^ Department of Clinical Laboratory Science, Chungbuk Health & Science University, Chungbuk 28150, Republic of Korea

^6^ Research Center, Dongnam Institute of Radiological and Medical Sciences, Busan 46033, Republic of Korea

***Correspondence:**

Man Hee Rhee, PhD

Laboratory of Physiology and Cell Signaling, College of Veterinary Medicine, Kyungpook National University, Daegu 41566, Republic of Korea

Tel: +82-53-950-5967; Fax +82-53-950-5955

Email address: rheemh@knu.ac.kr

**2. Methods**

***2.1. Chemicals and reagents***

Collagen (Native collagen fibrils, type I, from equine tendons), adenosine diphosphate (ADP), and thrombin were acquired from Chrono-log (Havertown, PA, USA). Fura-2-acetoxymethyl ester (fura-2/AM), dimethyl sulfoxide (DMSO), and ASA were purchased from Sigma-Aldrich (St. Louis, MO, USA). ATP Assay kit and Cyclic-AMP EIA kit were obtained from Biomedical Research Service Center (Buffalo, NY, USA) and Cayman Chemicals (Ann Arbor, MI, USA), respectively. Thromboxane-B2 ELISA kit was purchased from Enzo life sciences (USA). Fibrinogen Alexa Fluor 488 conjugate and Fibronectin adhesion assay kit were procured from Molecular Probes (Eugene, OR, USA) and Cell Biolabs Inc. (San Diego, CA, USA), respectively. Standard compounds (+)-catechin and catechin-7-*O*-β-_D_-apiofuranoside were purchased from ENSOL BIOSCIENCES INC. (Daejeon, Republic of Korea). Antibodies against Src, phospho-Src, extracellular signal-regulated kinases (ERK; p44/42), phospho-ERK (phospho-p44/42), stress-activated protein kinase (SAPK)/c-Jun N-terminal protein kinase (JNK), phospho-SAPK/JNK, p38^MAPK^, phospho-p38^MAPK^, phospho-PI3K (phospho-p85), PI3K (p85), Akt, phospho-Akt, vasodilator-stimulated phosphoprotein (VASP), phospho-VASP^ser157^, PKAαβγ, and β-actin were acquired from Cell Signaling Technology (Beverly, MA, USA). Commercial grade (+)-Catechin (Product No. ES090-A) and catechin-7-*O*-β-_D_-apiofuranoside (Product No. ES060-A) standards with ≥95% purity were purchased from Ensol Biosciences Inc. (Daejeon, Republic of Korea). Water was obtained from J. T. Baker (Phillipsburg, NJ, USA). All chemicals were of reagent grade.

***2.2. Preparation of washed human platelets***

Human platelet-rich plasma (PRP) collected from healthy volunteers who provided informed consent was obtained from the Korean Red Cross Blood Center (KRBC, Changwon, Korea), and washed platelet were prepared and adjusted to a final concentration of 5×10^8^/mL as previously described (Irfan et al., 2018a).

***2.3. Preparation of washed rat platelets***

Whole blood was collected from rats via heart puncture and anticoagulated with ACD solution. Blood was centrifuged at 170×*g* for 7 min to obtain PRP. Subsequently, PRP was centrifuged at 350×*g* for 7 min to isolate washed platelets. Platelet concentration was adjusted at 3×10^8^ cells/mL using Tyrode’s buffer (137 mM NaCl, 12 mM NaHCO_3_, 5.5 mM glucose, 2 mM KCl, 1 mM MgCl_2_, and 0.3 mM NaHPO_4_, pH 7.4) for use in platelet aggregation assays. All preparation procedures were performed at room temperature (23±2°C).

***2.4. Scanning electron microscope (SEM) analysis***

A field emission SEM (SU8220; Hitachi, Japan) was used to assess platelet shape change and aggregation by obtaining ultrastructure images. After platelet aggregation assay, the platelet mixture was treated with 0.5% paraformaldehyde (first fixation) and osmium tetroxide (second fixation), dehydrated with ascending concentrations of ethanol, freeze dried, and subsequently scanned as previously described (Irfan et al., 2018a).

***2.5. Assessment of ATP release and thromboxane-B2 production***

Washed platelets were pre-incubated with various concentrations of either *U. parvifolia* extract or vehicle for 1 min in the presence of 1 mM CaCl_2_ and subsequently stimulated with agonist for 5 min at 37°C with continuous stirring as previously described (Kim et al., 2017). Reaction was stopped and supernatant was obtained by centrifuging the platelet mixture; then, ATP secretion was measured on a luminometer (GloMax 20/20; Promega, Madison, WI, USA) using an ATP assay kit (Biomedical Research Service Center) and thromboxane-B2 production was assessed using TXB2 ELISA kit (Enzo Life Sciences, USA).

***2.6. Measurement of [Ca^2+^]_i_ mobilization***

Intracellular calcium ion concentration ([Ca^2+^]*_i_*) was assessed with Fura-2/AM as previously described (Jeong et al., 2017); PRP was incubated with 5 µM Fura-2/AM for 1 h at 37°C. Next, Fura-2/AM-loaded platelets were pre-incubated with either *U. parvifolia* extract or vehicle for 1 min in the presence of 1 mM CaCl_2_ and subsequently stimulated with collagen for 3 min. Fura-2 fluorescence in cytosol was measured using a spectrofluorometer (F-2500; Hitachi) and the following formula, as described by Schaeffer and Blaustein (Schaeffer and Blaustein, 1989): [Ca^2+^]*_i_* = 224 nM × (*F* − *F_min_*)/(*F_max_* − *F*), where 224 nM is the dissociation constant of the Fura-2-Ca^2+^complex, and *F_min_* and *F_max_* are the fluorescence intensities at very low and very high Ca^2+^ concentrations, respectively.

***2.7. Flow cytometry***

Fibrinogen binding in the presence or absence of *U. parvifolia* extract was measured via flow cytometry using Alexa Fluor 488‐human fibrinogen as previously described (Irfan et al., 2018b). Briefly, washed platelets were pre-treated with vehicle or various concentrations of *U. parvifolia* extract and stimulated with collagen for 5 min. Flow cytometric analysis was performed using a FACS Aria™ III flow cytometer® (Becton Dickinson Immunocytometry Systems, San Jose, CA, USA), and the data were analyzed using CellQuest software (Becton Dickinson Immunocytometry Systems).

***2.8. Fibronectin adhesion assay***

To determine the effect of *U. parvifolia* extract on platelet adhesion, a fibronectin adhesion assay was performed using the Fibronectin Adhesion Assay kit (Cell Biolabs, Inc.) according to the manufacturer’s protocol. Briefly, washed platelets pre-treated with vehicle, various extract concentrations, or GR155053 (α_IIb_β_3_ inhibitor) were incubated in fibronectin-coated wells for 90 min at 37°C and subsequently washed with DDW. Then, adhered cells were stained with crystal violet for 10 min and washed with DDW. Finally, the stained adhered cells were solubilized with extraction solution. The obtained lysate solution was transferred to a 96-well plate and its absorbance was measured at 540 nm.

***2.9. Clot retraction and kinetics of clot retraction***

PRP (250 µL) was mixed with red blood cells (5 µL) and incubated for 2 min with vehicle, various concentrations of *U. parvifolia* extract, or Y-27632 (ROCK inhibitor). Subsequently, Tyrode’s buffer was added to make the volume to 1 mL as previously described (Irfan et al., 2018b). Thrombin (1 U/mL) was added to initiate fibrin clot formation; then, clot retraction was observed for 2 h at room temperature and photographed with every 15 min intervals. Clot weight was measured as a marker of clot retraction. Kinetics of clot retraction were assessed as previously described (Misztal et al., 2013). Pictures were processed with Image-J software and clot surface areas were plotted as a percentage of clot retraction.

***2.10. Measurement of cyclic-AMP***

Washed platelets were pre-incubated with various concentrations of *U. parvifolia* extract, forskolin, or vehicle along with 1 mM CaCl_2_ for 1 min at 37°C, and then stimulated with collagen for 5 min in a platelet aggregometer. The reaction was terminated and the solution was centrifuged at 2000×*g* for 10 min at 4°C; furthermore, cAMP levels in the supernatant were determined using cAMP EIA kit (Cayman Chemical) according to the manufacturer’s protocol.

***2.11. Arteriovenous shunt model***

The antithrombotic activity of *U. parvifolia* extract was assessed in a rat extracorporeal shunt model as previously described (Irfan et al., 2018c). Rats were orally administered with the vehicle or *U. parvifolia* extract once daily for 3 days. Two hours after the last administration, rats were anesthetized with urethane (1.75 g/kg i.p.) and an incision was made over the trachea. The right jugular vein and left carotid artery were exposed, and the two ends of the extracorporeal shunt were inserted into them. The shunt consisted of two 12 cm long polyethylene tube (0.81 mm and 0.58 mm external and internal diameter, respectively), which is connected to a 6 cm long polyvinyl tube (3-mm internal diameter) by 5-mm silicone rubber plugs. A 6 cm long cotton thread was secured between the two plugs so that it remains longitudinally orientated in the blood flowing through the cannula. Before cannulation, the tube was filled with 0.9% saline solution. The shunt was left in place for 15 min after initiating extracorporeal circulation. Subsequently, blood flow was stopped, thread was removed, and the thrombus formed was separated from the thread and unclotted blood and weighed.

***2.12. In vivo bleeding assay***

Male mice were divided into three treatment groups (n=5, each). They were intraperitoneally administered with saline (control), ASA (50 mg/kg), or *U. parvifolia* extract (200 mg/kg) once daily for 3 days. One hour after the last administration, mice were anaesthetized, and tail bleeding assay was performed as previously described (Irfan et al., 2018b). Briefly, the tail was pre-warmed for 3 min in a 0.9% saline solution at 37°C. Bleeding was induced by precisely transecting the mouse tail at 5 mm from the tip. The distal portion of the tail (3 cm) was vertically immersed into 0.9% saline solution at 37°C. The time from transection initiation to bleeding cessation was recorded as the bleeding time.

**References**

Irfan, M., Jeong, D., Kwon, H.-W., Shin, J.-H., Park, S.-J., Kwak, D., et al. (2018a). Ginsenoside-Rp3 inhibits platelet activation and thrombus formation by regulating MAPK and cyclic nucleotide signaling. *Vascular pharmacology* 109**,** 45-55.

Irfan, M., Jeong, D., Saba, E., Kwon, H.-W., Shin, J.-H., Jeon, B.-R., et al. (2018b). Gintonin modulates platelet function and inhibits thrombus formation via impaired glycoprotein VI signaling. *Platelets***,** 1-10.

Irfan, M., Kwon, T.-H., Yun, B.-S., Park, N.-H., and Rhee, M.H. (2018c). Eisenia bicyclis (brown alga) modulates platelet function and inhibits thrombus formation via impaired P2Y12 receptor signaling pathway. *Phytomedicine* 40**,** 79-87.

Jeong, D., Irfan, M., Kim, S.-D., Kim, S., Oh, J.-H., Park, C.-K., et al. (2017). Ginsenoside Rg3-enriched red ginseng extract inhibits platelet activation and in vivo thrombus formation. *Journal of ginseng research* 41(4)**,** 548-555.

Kim, D.S., Irfan, M., Sung, Y.Y., Kim, S.H., Park, S.H., Choi, Y.H., et al. (2017). Schisandra chinensis and Morus alba Synergistically Inhibit In Vivo Thrombus Formation and Platelet Aggregation by Impairing the Glycoprotein VI Pathway. *Evid Based Complement Alternat Med* 2017**,** 7839658. doi: 10.1155/2017/7839658.

Misztal, T., Przesław, K., Rusak, T., and Tomasiak, M. (2013). Peroxynitrite–altered platelet mitochondria—A new link between inflammation and hemostasis. *Thrombosis research* 131(1)**,** e17-e25.

Schaeffer, J., and Blaustein, M.P. (1989). Platelet free calcium concentrations measured with fura-2 are influenced by the transmembrane sodium gradient. *Cell Calcium* 10(2)**,** 101-113.
